# Time-restricted feeding ameliorates dextran sulfate sodium-induced colitis *via* reducing intestinal inflammation

**DOI:** 10.3389/fnut.2022.1043783

**Published:** 2022-12-23

**Authors:** Shuo Song, Lingling Chen, Meijuan Bai, Shuo Wang, Xiaoyi Ye, Yijun Lin, Xuemei Luo, Zixuan Li, Lingling Zhang, Xinyu Zhu, Zinan Wang, Yan Chen

**Affiliations:** ^1^Key Laboratory of Nutrition, Metabolism and Food Safety, Shanghai Institute of Nutrition and Health, University of Chinese Academy of Sciences, Chinese Academy of Sciences (CAS), Shanghai, China; ^2^School of Life Science and Technology, ShanghaiTech University, Shanghai, China

**Keywords:** inflammatory bowel disease, time-restricted feeding, intestine, inflammation, T cells

## Abstract

Time-restricted feeding (TRF) is an emerging dietary intervention that improves metabolic disorders such as obesity, insulin resistance and dyslipidemia. Inflammatory bowel disease (IBD) is a chronic inflammatory disorder affecting the gastrointestinal tract, where nutrition plays an important role in its pathogenesis. Although numerous strategies of nutritional intervention have been reported, whether TRF can improve IBD has been elusive. In this study, we investigated the effect of two cycles of 7-day TRF intervention in a dextran sulfate sodium-induced IBD mouse model. We found that TRF was able to reduce the disease activity index and ameliorate the IBD-associated symptoms, as well as increase the number of colonic crypts and decrease the histological score in the colon. Furthermore, TRF lowered the percentage of CD4^+^ T cells in the peripheral blood and mesenteric lymph node, and increased the number of CD4^+^CD25^+^ T cells in the mesenteric lymph nodes. Additionally, TRF reduced the infiltration of leukocytes and macrophages around the crypt base in the colon. However, unlike the intermittent caloric restriction with fasting-mimicking diet, TRF was not able to increase the markers of progenitor and cell proliferation in the colon. Collectively, these results demonstrated that TRF is able to improve IBD in mice via reduction in intestinal inflammation.

## Introduction

Inflammatory bowel disease (IBD) has become a global healthcare problem with a continuously increasing incidence (1). Crohn’s disease and ulcerative colitis are the two main components of IBD. Although the etiology of IBD remains unknown, numerous evidences suggest that genetics, environmental factors, intestinal microbiota, innate immunity, and adaptive immune are all involved in the pathogenesis of IBD (2–4). On the other hand, intermittent fasting (IF) is a form of dietary restriction (DR) that alternating the periods of eating and fasting, which comprise alternate-day fasting, 5:2 diet, fasting-mimicking diet (FMD), and time-restricted feeding (TRF) (5). Studies based on animal models and clinical trials revealed that IF can reduce body weight, improve insulin sensitivity, lower blood pressure and decrease the markers of inflammation and oxidative stress (6–10). TRF is a type of IF which becomes increasingly popular for its consistent improvement in health by maintaining a daily cycle of feeding and fasting to support robust circadian rhythms (11). On the one hand, circadian rhythms regulate the endocrine system and autonomic nervous system (12). In addition, nutrient metabolism contributes to the physiological and metabolic homeostasis which is associated with the homeostasis of immune system (11, 12). Disruption of circadian rhythm, on the other hand, can increase the risk of metabolic syndrome which is comprised of the components of obesity, insulin resistance, hypertension, dyslipidemia, and chronic inflammation (13, 14). Recent studies have indicated that TRF improves insulin sensitivity and reduces body weight, blood pressure, atherogenic lipids and oxidative stress in patients with metabolic syndrome, while prevents obesity and metabolic syndrome in mice lacking a circadian clock (5, 15–17).

We and others have demonstrated that intermittent application of a fasting-mimicking diet can improve IBD in mice (18, 19). However, whether or not TRF can improve IBD is unknown. To address this issue, we designed an experiment to investigate the effect of TRF in the dextran sulfate sodium (DSS)-induced IBD mouse model. Our results demonstrate that two cycles of 7-day TRF is able to ameliorate IBD-associated symptoms and reduce the systemic and intestinal inflammation in mice.

## Materials and methods

### Mouse model and diets

All animal experimental procedures were approved by the Institutional Animal Care and Use Committee (IACUC) guidelines of Shanghai Institute of Nutrition and Health, Chinese Academy of Sciences with an approval number SINH-2020-CY-1. 6-week-old female C57BL/6J mice were purchased from the SLAC company (Shanghai, China) and group-housed for three weeks in 12h:12h light-dark cycle. All mice were maintained in a pathogen-free environment and kept in clear cages with constant temperature and humidity. Mice were randomly assigned to control group, DSS + FMD group and DSS + TRF group. During the dietary cycle, all mice were provided with 2.5% w/w DSS (MP Biomedicals, Santa Ana, CA, USA) as the only drinking source for 5 days, then water for 9 days. The normal chow was purchased from Shanghai Pu Lu Teng Biological Technology Co., Ltd. (Shanghai, China) and the nutrient composition is 21.6% protein, 51.4% carbohydrate, 4.5% fat, and 3% dietary fibers. The FMD (Gembynear Nutrition Bar or Zhenbainian in Chinese) used in this study were provided by the Beijing Winlife Research Institution of Nutrition, Health, Food Science, and Technology (Beijing, China). Every 100 g of the bar contains 401.8 kcal of calorie, 44.3 g of carbohydrate, 17.8 g of protein, 14.2 g of fat, and 12.5 g of dietary fibers (19). The nutrient composition of the FMD is 17.8% protein, 44.3% carbohydrate, 14.2% fat, and 12.5% dietary fibers. The mice in the DSS + FMD group were kept separately to ensure the calorie intake of each individual in the DSS + FMD group matched 30% of that in the control group during the 3-day FMD administration. The mice in DSS + TRF group had access to food with normal chow *ad libitum* for 6 h during the dark phase then fasting for the rest of the time in TRF period. The method of TFR was based on recent publications by other groups (16, 20). The establishment of the DSS mouse model was previously reported by our group (19).

### Disease activity index scoring

Disease activity index was used to evaluate the severity of IBD, it comprises three parts: the body weight loss, stool consistency and blood in stools. The DAI scores were recorded from the first day of cycle 2 to the last day of cycle 3. The criterion of body weight loss scores as follows: score 0 for no body weight loss, score 1 for body weight loss within 1-5%, score 2 for body weight loss within 5-10%, score 3 for body weight loss within 10-20% and score 4 for body weight loss more than 20%. Stool consistency scores as follows: score 0 for solid pellets, score 1 for soft but adherent in pellet shape, score 2 for loose stool but with some solidity, score 3 for loose stool with signs of liquid consistency and score 4 for diarrhea. The visible blood in the stools and rectum was directly recorded and the occult blood in stools was tested by occult blood test strip (W.H.P.M. Bioresearch & Technology Co., Beijing, China). Stool consistency DAI scores as follows: score 0 for hemoccult negative, score 1 for hemoccult positive, score 2 for hemoccult positive with visual pellet bleeding, score 3 for hemoccult positive with visual pellet and rectal bleeding and score 4 for hemoccult positive with gross visual pellet and rectal bleeding.

### H&E staining and colon histological score

Proximal colon tissues were dissected from the mice for histological analyses. The tissue sample were fixed in 4% paraformaldehyde for more than 24 h at room temperature, dehydrated and embedded in paraffin. The embedded colon tissues were sliced to the thickness of 4 μm. The sections were stained by Hematoxylin & Eosin staining Kit with the manufacture’s protocol (Servicebio, Wuhan, Hubei, China) and the images was captured by an optical microscope (Olympus, Japan). The severity of inflammation and damage in the colon tissue is evaluated by histopathological scoring in the H&E stain colon sections. The histological scores were measured as follows: (0) For no inflammation and damage in the mucosa of colon; (1) For mild edema and inflammation in the mucosa with 1/3 crypts disappeared; (2) For moderate inflammation in the mucosa with 2/3 crypts disappeared; (3) For moderate inflammation in the mucosa with crypts disappeared completely; and (4) For serious damage and destruction in the mucosa of colon.

### Flow cytometry

The immune cells were isolated from the peripheral blood, spleen and mesenteric lymph nodes. The lymphocytes of peripheral blood were separated by centrifugation and the erythrocytes were lysed with lysis buffer (BD Bioscience, San Diego, CA, USA). The isolated spleen and mesenteric lymph node were incubated in PBS, then ground and filtered through a 70 μm filter, followed by cell collection via centrifugation. The erythrocytes in the spleen were lysed with lysis buffer (BD Bioscience). The antibodies used were APC-cyTM7 hamster anti-mouse CD3e (BD Biosciences, New Jersey, USA), PE rat anti-mouse CD4 (BD, Biosciences), PE-CyTM rat anti-mouse CD8a (BD Biosciences) and FITC CD25 (BD, Biosciences). The cells were firstly stained with live-dead BV510 dye (BD, Biosciences) for 20 min to exclude the dead cells, followed by washing with PBS and resuspension in PBS containing CD3e, CD4, CD8, and CD25 antibodies for 30 min. The cells were finally washed with PBS and resuspended in 400 μl PBS. The flow cytometric analysis of T cells was based on gating. Firstly, the live cells were gated by BV510 negative. Secondly, the CD3 positive cells were gated to distinguish the T cells. After that, the CD4 and CD8 were gated to differentiate subsets of T cells. Finally, the CD25 was gated based on the CD4-positive cells. The samples were analyzed on a CytoFLEX LX cytometer (Beckman Coulter, Life Sciences, Indianapolis, USA) and data were captured with FlowJo software.

### Immunofluorescence analysis

The paraffin sections of colon tissues were deparaffinized in xylene and rehydrated in gradient ethanol (100, 90, 70, 50, and 30%) for 5 min each concentration, then washed with distilled water. The antigen was retrieved in 0.1 M citrate buffer (pH 6.0), maintained at a sub-boiling temperature for 30 min and then cooled to room temperature. The slides were blocked with blocking buffer (PBS + 3% goat serum + 0.1% triton-100) for 1.5 h at room temperature. The slides were then incubated with primary antibodies (diluted with PBS) overnight at 4°C in a wet box. The primary antibodies used were as follows: rabbit anti-CD45 (1:200, Cell Signaling Technology, Boston, MA, USA), rabbit anti-F4/80 (1:200, Cell Signaling Technology), rabbit anti-Lgr5 (1:200, Abcam, MA, USA), mouse anti-PCNA (1:2,000, Cell Signaling Technology) and mouse anti-ki67 (1:50, BD Biosciences). The slides were rinsed three times with PBS and incubated with secondary antibodies (diluted with PBS) at room temperature for 1h in a dark box. The secondary antibodies used for the immunofluorescent staining were as follows: Alexa Fluor 488 goat anti-rabbit IgG (1:500, Life Technologies, Eugene, OR, USA), Alexa Fluor 488 goat anti-mouse IgG (1:500, Life Technologies), Alexa Fluor 546 goat anti-rabbit IgG (1:500, Life Technologies) and Alexa Fluor 546 goat anti-mouse IgG (1:500, Life Technologies). The slides were then washed three times with PBS and the nuclei were stained with Hoechst 33,342 (Molecular Probes, Eugene, OR, USA). Finally, the slides were covered with anti-fading aqueous mounting medium (Sigma-Aldrich, Saint Louis, MO 63103, USA). Images were captured at 20 X with an LSM 510 confocal microscope (Zeiss, Jena, Germany).

### Statistical analysis

Student’s *t*-test was used for pair-wise comparisons, one-way ANOVA was used for more than two groups. GraphPad Prism v.8 was used for statistical analysis. All data was presented as mean ± SEM. *p* values of <0.05 were considered statistically significant.

## Results

### Two TRF cycles ameliorate IBD-associated symptoms in a DSS-induced IBD mouse model

**FIGURE 1:**
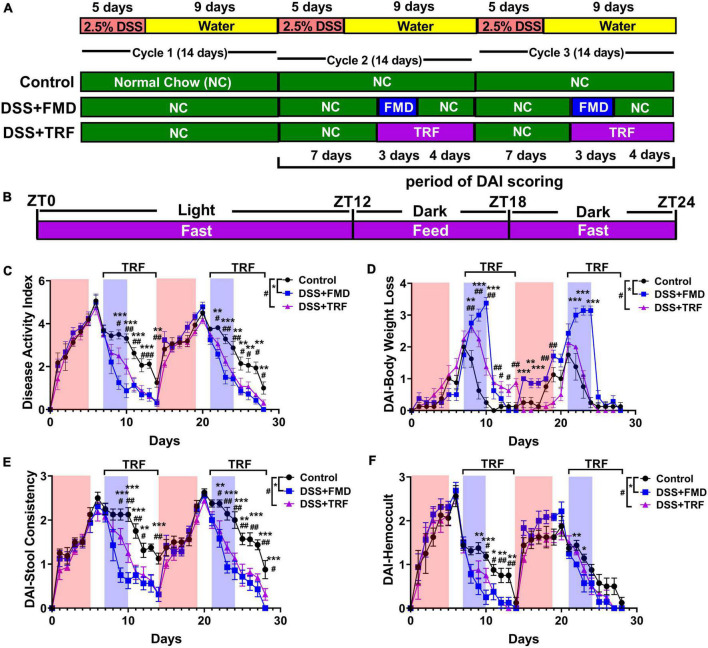
TRF reduces disease activity index (DAI) and ameliorates IBD-associated symptoms in mice. **(A)** The schematic diagram of the experimental design. **(B)** The schematic diagram of TRF diet for each day during the intervention. **(C)** Cumulative DAI scores (excluding body weight loss score) that starting at the first day of cycle 2 (*n* = 8 for each group). **(D)** DAI scores of body weight loss. **(E)** DAI scores of stool consistency. **(F)** DAI scores of hemoccult test. In **(C–F)**, the red shade denotes the duration of DSS treatment and the blue shade indicates the duration of FMD intervention, the duration of TRF is represented by the black bracket. Data are presented as mean ± SEM. The * sign represents the differences between the DSS + FMD group and the control group, and the # sign represents the differences between the DSS + TRF group and the control group (1 sign for *P* < 0.05, 2 signs for *P* < 0.01, 3 signs for *P* < 0.001).

To explore the potential effect of TRF on IBD, we used DSS to induce ulcerative colitis in mice, a commonly used mouse model (21). DSS is a sulfated polysaccharide that is toxic to the intestinal epithelium and causes intestinal barrier impairment in mice, shown as IBD-associated symptoms such as weight loss, diarrhea, and bloody stool (21, 22). In our experiment, the mice were treated with 2.5% DSS in drinking water for 5 days to induce ulcerative colitis, followed by 9 days of no treatment in each cycle. Three cycles were carried out in total (Figure 1A). Female C57BL/6 mice at 9 weeks old were randomly divided into 3 groups. The control group was fed with normal chow *ad libitum* throughout the experiment (Figure 1A). As a positive control, the DSS + FMD group was administered with 2 cycles of FMD intervention (Figure 1A). We previously found that intermittent application of the FMD could ameliorate IBD in mice by reducing systemic and intestinal inflammation, while promoting the repair and regeneration of intestinal epithelium (19). For the FMD intervention group, FMD was administered for 3 days starting at the 8th day of cycle 2 and cycle 3, followed by normal chow *ad libitum* for 4 days (Figure 1A). The calorie intake of mice in the DSS + FMD group was 30% of the control group. The DSS + TRF group contained 2 cycles of TRF intervention starting from the 8^th^ day of cycle 2 and cycle 3 (Figure 1A). During the TRF intervention, mice were fed with normal chow *ad libitum* starting at 18:00 [Zeitgeber time 12 (ZT12)] and ending at 24:00 [Zeitgeber time 18 (ZT18)] with a total of 6 h of feeding time, and the other 18 h was in the state of fasting for each day (Figure 1B).

The disease activity index (DAI) was measured every day for the three groups of mice from the beginning of cycle 2 (Figures 1C-F). In general, the score of DAI consists of weight loss, stool consistency and hemoccult. However, as caloric restriction was performed in the DSS + FMD group, we modified DAI scores by only including those of stool consistency and hemoccult test. Both the DSS + TRF and DSS + FMD groups showed a significant decrease in DAI scores comparing to the control group from the second day of TRF or FMD onward. The reduction of DAI scores by FMD and TRF persisted until the end of cycle 2 (Figure 1C). Such reduction of DAI by FMD and TRF was also seen in cycle 3 of DSS treatment (Figure 1C). In addition, DAI scores based on body weight revealed that the DSS + TRF group performed better than the control group (Figure 1D). In addition, both the DSS + TRF and DSS + FMD groups had significant decrease in the DAI scores of stool consistency and hemoccult comparing to the control group at multiple time points (Figures 1E–F). Together, these data clearly indicated that TRF is able to ameliorate IBD-associated symptoms in the DSS-induced IBD mouse model. To exclude the possibility that TRF might incur caloric restriction which itself might improve IBD, we monitored daily food and water intake of the mice starting at the first day of cycle 2 and found that there was no significant difference between the DSS + TRF group and the control group (Supplementary Figure 1). This indicates that the administration of TRF improves IBD, yet not through the altering of caloric intake in the mice. In addition, by comparing the two diet-restricted groups, it was found that the FMD group performed slightly better than the TRF group in improving DAI (Supplementary Figure 2).

### TRF increases the number of crypts and reduces histological score in the colon

**FIGURE 2:**
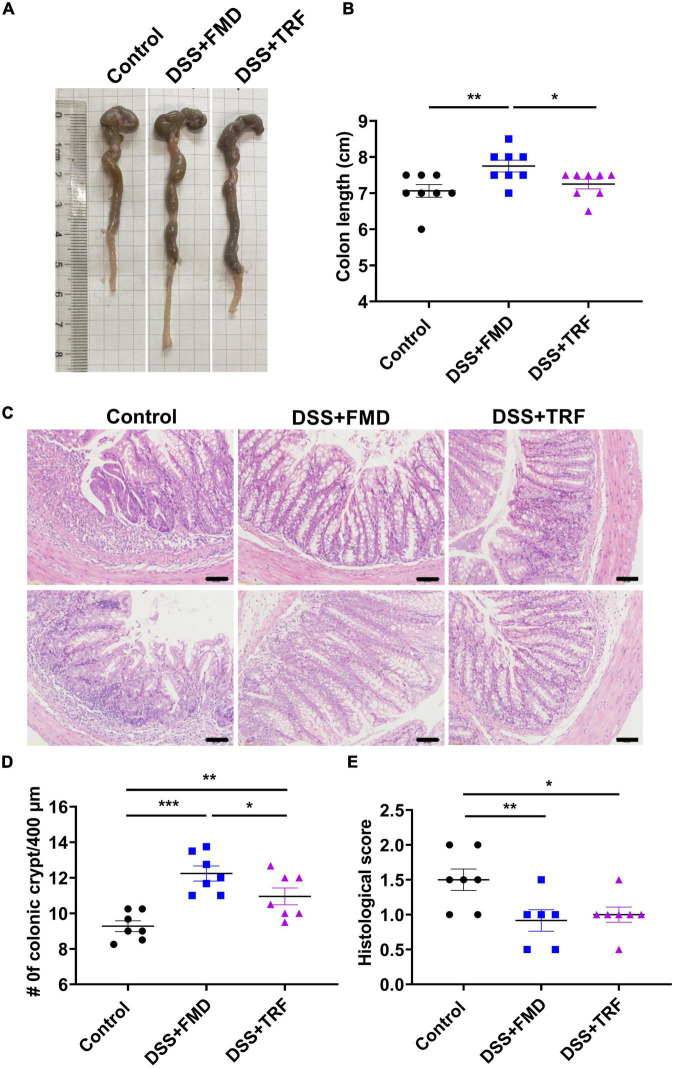
TRF increases the number of crypts and reduce the histological score in the colon. **(A)** Representative images of the colon at the end of the experiment. **(B)** The length of colon of the mice (*n* = 8 for each group). **(C)** Histology of colon tissue examined by HE staining. The images were taken at 20 X magnification. Scale bars: 50 μm. **(D)** Changes of colonic crypts number of the mice (*n* = 7 for each group). **(E)** Histological scores of colon tissues based on HE staining (*n* = 6 to 7 for each group). Data are presented as mean ± SEM; *P < 0.05, **P < 0.01, ***P < 0.001.

After the DSS treatment, the length of the colon is shortened due to intestinal damage and inflammation. Thus, we measured the colon length among the three groups of the mice (Figure 2A). After 3 DSS cycles, we observed that the average colon length of the control group was 7.06 cm whereas it was 7.75 cm in the DSS + FMD group, which was significantly longer than that of the control group (Figure 2B). However, the average colon length of the DSS + TRF group was 7.25 cm, not significantly different from the control group (Figure 2B). In addition, we measured the length of small intestine and there was no significant difference among the three groups (data not shown).

The crypt is a gland in the intestine, which contains multiple types of cells such as goblet cells, Paneth cells and intestinal stem cells. The crypt plays an important role in maintaining the normal physiological function of the intestine (23). DSS treatment could destroy the colonic crypt structure so that the number of crypts can reflect the integrity of the intestinal epithelium. After DSS treatment, there were multiple features of colitis in the colon such as loss of crypts, infiltration of immune cells and ulceration (Figure 2C). We counted the number of crypts with the colonic tissue and found that the amount of the crypts in both the DSS + TRF and DSS + FMD groups were significantly increased in comparison with the control group (Figure 2D). Furthermore, pathological scores in both the DSS + TRF and DSS + FMD groups were significantly lowered (Figure 2E). Taken together, the increase in crypt number and decrease in histopathological score in the DSS + TRF group indicated that TRF can reduce the DSS-induced damage in the colon.

### TRF alters T cell profile in peripheral blood and mesenteric lymph node

**FIGURE 3:**
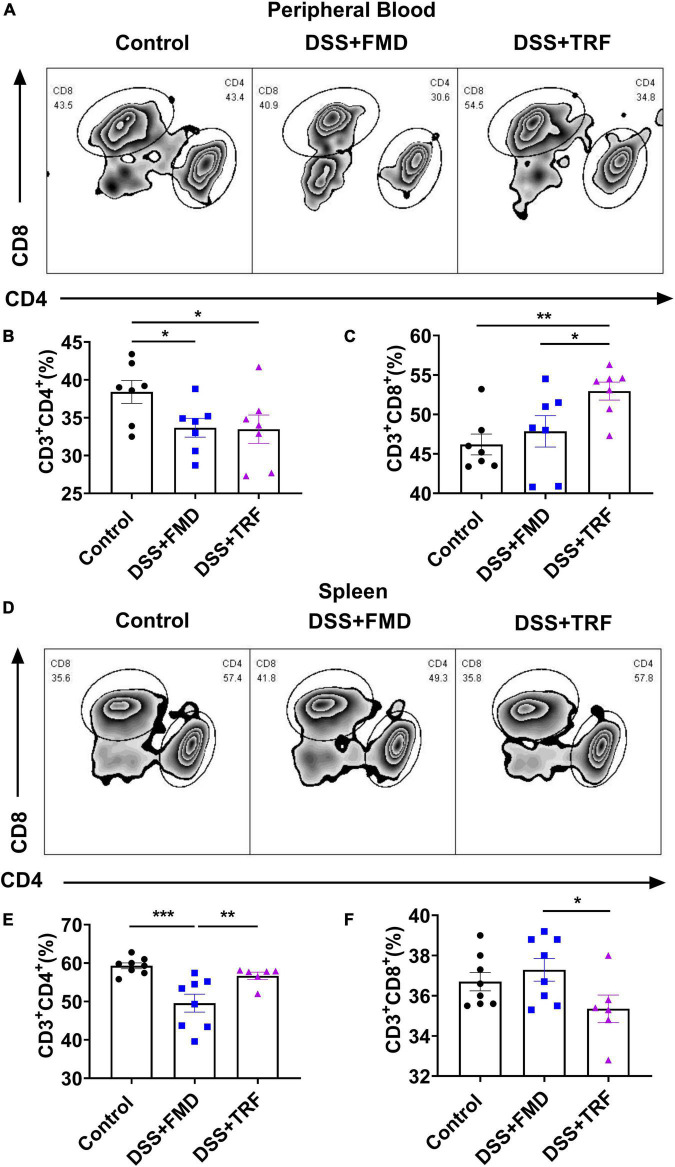
TRF alters T cells profile in the peripheral blood and spleen. **(A)** Results of flow cytometry analysis to detect the proportion changes of CD4^+^ and CD8^+^ T cells in peripheral blood of mice at the end of experiment (*n* = 7 for each group). **(B)** Percentage of CD4^+^ T cells in the peripheral blood. **(C)** Percentage of CD8^+^ T cells in the peripheral blood. **(D)** Results of flow cytometry analysis to detect the CD4^+^ and CD8^+^ T cells in the spleen at the end of experiment (*n* = 6 to 8 for each group). **(E)** Percentage of CD4^+^ T cells in the spleen. **(F)** Percentage of CD8^+^ T cells in the spleen. Data are presented as mean ± SEM; *P < 0.05, **P < 0.01, ***P < 0.001.

Both CD4^+^ and CD8^+^ T cells are important components of the adaptive immune. It has been indicated that the levels of circulating CD4^+^ and CD8^+^ T cells are associated with IBD(1). We detected the proportions of CD4^+^ and CD8^+^ T cells in the peripheral blood and spleen by flow cytometry to evaluate the severity of systemic inflammation in the IBD mouse model (Figures 3A, D). After two cycles of TRF and FMD intervention, the percentage of CD4^+^ T cells in peripheral blood in both the DSS + TRF and DSS + FMD groups were significantly decreased as compared to the control groups (Figure 3B). There was no significant difference in the percentage of CD8^+^ T cells between the DSS + FMD group and the control group in peripheral blood, while the CD8^+^ T cells in the DSS + TRF group were significantly increased (Figure 3C). On the other hand, in the spleen, the percentage of CD4^+^ T cells in the DSS + FMD group is lower than that of the control group, while there was no significant difference between the DSS + TRF group and control group (Figure 3E). For CD8^+^ T cells in the spleen, we did not find significant difference between the DSS + TRF group and the control group (Figure 3F). Overall, these data suggested that administrating two cycles of TRF is able to decrease the percentage of CD4^+^ T cells in the peripheral blood, likely contributing to the reduction of systemic inflammation by TRF.

**FIGURE 4:**
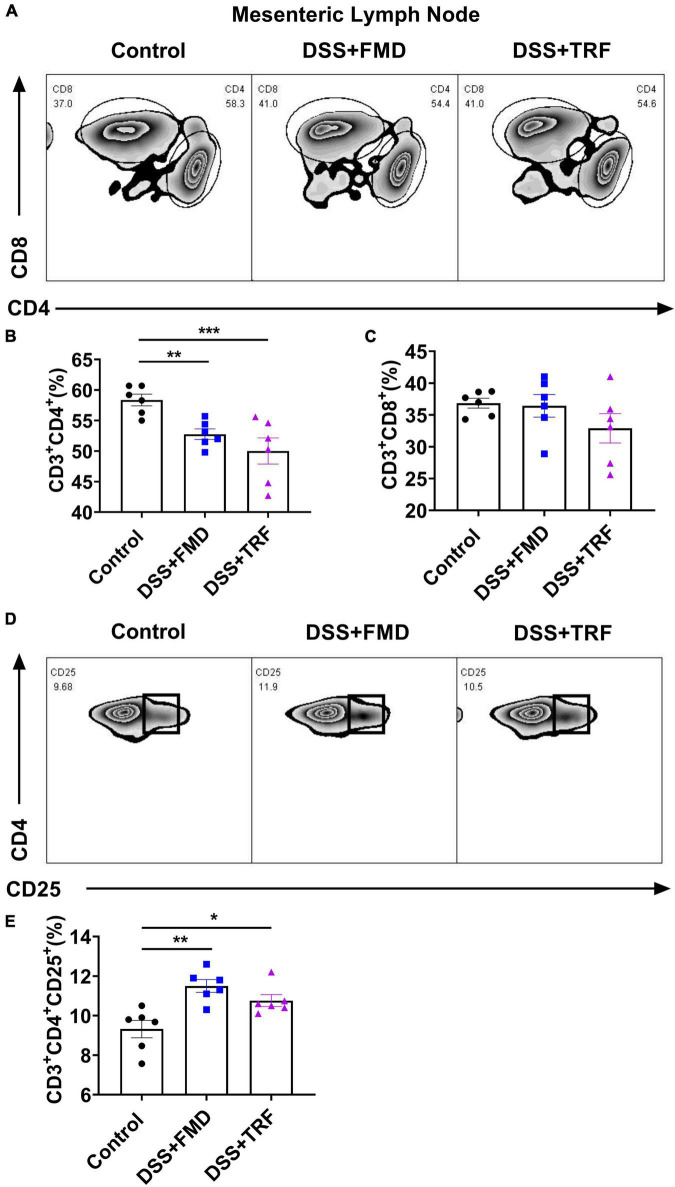
TRF alters the T cells profile in the mesenteric lymph nodes. **(A)** Results of flow cytometry analysis for CD4^+^ and CD8^+^ T cells in the mesenteric lymph nodes at the end of experiment (*n* = 6 for each group). **(B)** Percentage of CD4^+^ T cells in the mesenteric lymph nodes. **(C)** Percentage of CD8^+^ T cells in the mesenteric lymph nodes. **(D)** Results of flow cytometry analysis of CD4^+^CD25^+^ T cells in the mesenteric lymph nodes (*n* = 6 for each group). **(E)** Percentage of CD4^+^CD25^+^ T cells in the mesenteric lymph nodes. Data are presented as mean ± SEM; *P < 0.05, **P < 0.01, ***P < 0.001.

The mesenteric lymph nodes are located in the walls of the intestine and their function is to continually filter the lymph fluid. The mesenteric lymph nodes are important components of the immune system because microorganisms such as bacteria and viruses could be trapped there so that mesenteric lymph nodes would protect the intestine from the invasion of pathogenic microorganisms (24). As the profile of T cells in mesenteric lymph nodes reflects the degree of inflammation in the intestine, we used flow cytometry to analyze T cell profile in the mesenteric lymph nodes (Figure 4). As for CD4^+^ T cells, there was a significant decrease in both the DSS + TRF and DSS + FMD groups as compared to the control group (Figure 4B), while no significant difference was found in the percentage of CD8^+^ T cells among the three groups (Figure 4C). Additionally, regulatory T cells (Treg cells) play an important role in modulating the immune system, maintaining tolerance to self-antigens and preventing the autoimmune disease. CD25 is a highly expressed marker in Treg cells (25). In mesenteric lymph nodes, the percentage of CD4^+^CD25^+^ T cells in both DSS + TRF and DSS + FMD groups were significantly increased in comparison with the control group (Figure 4E). These results thus indicated that the TRF can reduce the percentage of CD4^+^ T cells and increase the percentage of CD4^+^CD25^+^ T cells in the mesenteric lymph nodes.

### TRF reduces the infiltration of leukocytes and macrophages around the crypt base in the lamina propria of colon

**FIGURE 5:**
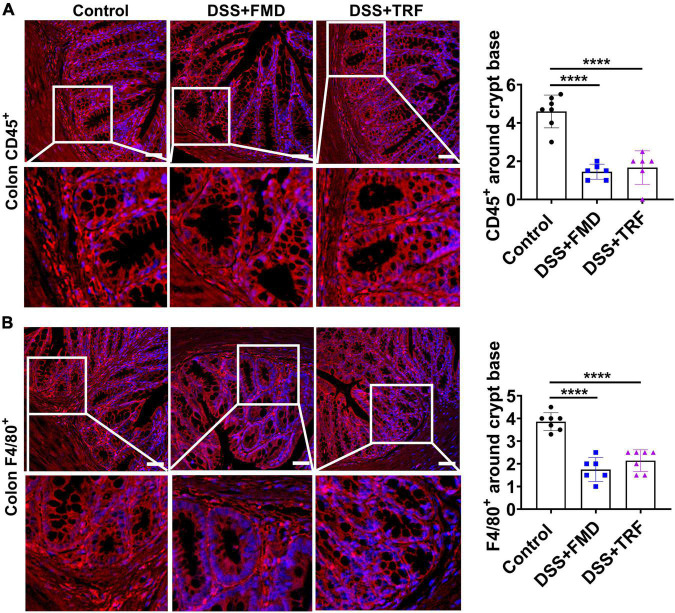
TRF reduces the infiltration of leukocytes and macrophages around the crypt base in the lamina propria of colon. **(A)** Representative images of CD45^+^ immunofluorescent staining (red color) using colon sections. The nuclei were stained with DAPI (blue color). Quantitation of CD45^+^ cells around the crypt base is shown on the right (*n* = 6 to 7 for each group). **(B)** Representative images of F4/80^+^ immunofluorescent staining (red color) in the colon. The nuclei were stained with DAPI (blue color). Quantitation of F4/80^+^ cells around the crypt base is shown on the right (*n* = 6 to 7 for each group). Data are presented as mean ± SEM; ****P < 0.0001. The images were taken at 20 X magnification. Scale bar: 50 μm.

The lamina propria (LP) is a large layer of connective tissue which separates the innermost layer of epithelial cells from smooth muscle tissue of the intestine. During the epithelium breakdown or pathogen infection, the immune cells are activated and the inflammatory cells infiltrate the LP to kill and clear invading microbes. Therefore, the infiltration of immune cells around the crypts in LP is closely associated with the severity of inflammation in the intestine. In order to evaluate the severity of inflammation in the colon, we detected inflammatory markers in the paraffin section of the colon tissue by immunofluorescence (Figure 5). Leukocytes are the major cellular components of the inflammation with CD45 as a marker for leukocytes. We found that the number of CD45^+^ cells around the crypt base in the LP of the DSS + TRF and DSS + FMD groups was significantly decreased as compared to the control group (Figure 5A). Macrophages are the key components of the innate immune system which are formed in response to infection, tissue damage or dead cells. Therefore, we also analyzed the expression of F4/80^+^, a major marker for macrophages. We observed a significant reduction in the number of F4/80^+^ cells surrounding the crypt base in both the DSS + TRF and DSS + FMD groups in comparison with the control group (Figure 5B). Overall, these results indicated that TRF can reduce the infiltration of leukocytes and macrophages around the crypt base in the LP of the colon, which further proved that TRF is able to ameliorate local inflammation of IBD in the mouse model.

### TRF cannot increase regeneration of intestinal epithelium, different from FMD

**FIGURE 6:**
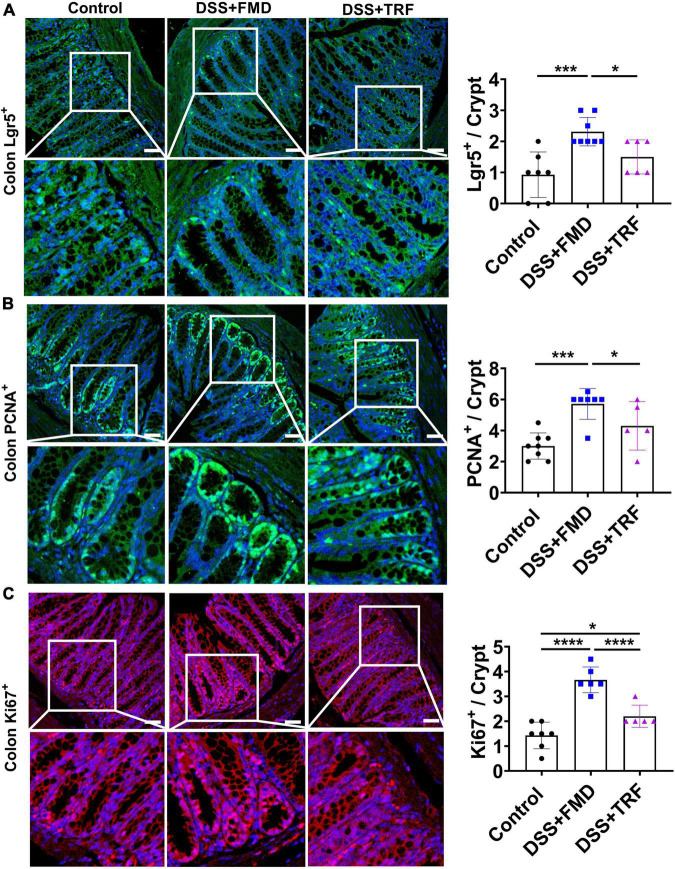
FMD but not TRF increases the markers of progenitor and proliferating cells in colon. **(A)** Representative images of Lgr5^+^ immunofluorescent (IF) staining in the colon (red color). Quantitation of Lgr5^+^ cells in the crypt of colon is shown on the right (*n* = 6 to 8 for each group). **(B)** Representative images of PCNA^+^ IF staining in the colon. Quantitation of PCNA^+^ cells in the crypt of colon is shown on the right (*n* = 5 to 8 for each group). **(C)** Representative images of Ki67^+^ IF staining in the colon. Quantitation of Ki67^+^ cells in the crypt of colon is shown on the right (*n* = 5 to 7 for each group). Data are presented as mean ± SEM; *P < 0.05, ***P < 0.001, ****P < 0.0001. IF images were taken at 20 X magnification. Scale bar: 50 μm.

Previously, it has been demonstrated that the intermittent administration of FMD can elevate the regeneration of intestinal epithelium (18, 19). We measured the number of Lgr5^+^ cells, PCNA^+^ cells and Ki67^+^ cells in the crypt of colon (Figure 6). Lgr5 is a marker of the intestinal stem cells (ISCs), while PCNA and Ki67 are the markers of cell proliferation (26–28). We found that the numbers of Lgr5^+^ cells, PCNA^+^ cells, and Ki67^+^ cells per crypt were all significantly increased in the DSS + FMD group as compared to the control group (Figure 6). Whereas the numbers of these cells in the DSS-TRF group were not significantly altered (Figure 6). These results thus suggested that being different from intermittent caloric restriction using FMD, TRF has no effect on the self-renewal of ISCs or regeneration of intestinal epithelium.

## Discussion

To our knowledge, this is the first report showing that TRF can improve IBD in a mouse model. Our study has demonstrated the effectiveness of two-cycle-TRF in reducing the DAI score and ameliorating the symptoms associated with IBD in the DSS-induced ulcerative colitis mouse model. According to histological analysis, TRF amends the degree of damage and inflammation in the colon, while increases the number of crypts at the same time. TRF reduced CD4^+^ T cells in the peripheral blood, which indicates a reduction in systemic inflammation by TRF. Additionally, TRF reduced CD4^+^ T cells and increased CD4^+^CD25^+^ cells in mesenteric lymph nodes. As CD4^+^CD25^+^ T cells are mainly Treg cells that play an important role in the immune system, TRF-mediated increases in these cells are likely to underlie its modulatory effect on local inflammation in our model. Furthermore, we found that the infiltration of leukocytes and macrophages around the crypt base in the colon was significantly decreased by TRF, providing further evidence that TRF may reduce the activation or recruitment of immune cells in the intestine. Taken together, these results indicated that two cycles of 7-day TRF can ameliorate the IBD-associated symptoms and reduce systemic and intestinal inflammation in the DSS-induced IBD mouse model. In addition, it was recently discovered that intestinal epithelial lymphocytes (IEL) are a group of unique T cells important in modulating intestinal inflammation (29) and it is an issue worth further investigation in our model.

Our study also revealed that TRF is somewhat different from the intermittent caloric restriction via FMD in improving IBD. Recently, our group reported that intermittent caloric restriction using FMD can reduce systemic and intestinal inflammation, and promote the regeneration and repair of the intestinal epithelium to ameliorate IBD in the mice (19). In this study, we included a DSS + FMD group as a positive control to compare it to the DSS + TRF group side-by-side. Interestingly, the reduction of the DAI score, stool consistency DAI score and hemoccult DAI score in the DSS + TRF group were very similar to the DSS + FMD group. However, while the shortening of colon length was reversed by FMD, TRF did not affect the shortening of colon length. Both TRF and FMD increased the number of colonic crypts and reduced the histological score. In addition, both TRF and FMD reduced the percentage of CD4^+^ T cells in the peripheral blood and mesenteric lymph nodes, indicating that both TRF and FMD can reduce systemic and local inflammation. Consistently, both TRF and FMD decreased the infiltration of leukocytes and macrophages around the crypt base in the colon. However, TRF but not FMD elevated the percentage of CD8^+^ T cells in the peripheral blood. It was previously reported that CD8^+^ T cells comprise the cytotoxic CD8^+^ T cells (Tc 1), IL-17-producing CD8^+^ T cells (Tc17), regulatory CD8^+^ T cells, and tissue-resident memory CD8^+^ T cells (Trm) (30). Both Tc1 and Tc17 cells are considered to contribute to the pathogenesis of IBD, while the subsets of regulatory CD8^+^ T cells and Trm cells exhibit anti-inflammatory activities (30). Thus, it will be of interest to investigate the immune-modulatory effects of CD8^+^ T cells by TRF in the future.

It is notable that previous studies by others also reported that intermittent CR could improve IBD (18, 31). In terms of the FMD group used in our study, Rangan’s study used much lower calorie intake than ours (18). During FMD administration, they used 50% calorie intake on the first day and 10% calorie intake in the following three days with an average of 20% calorie intake per day. While in our study, we used 30% calorie intake for three consecutive days in our FMD group, which is also different from the ones reported in Okada’s report (31). Another major difference between Rangan’s study and ours is the composition of FMD. The FMD used in Rangan’s study was low in carbohydrate and protein, and high in fat (18), but the FMD used in our study was low in carbohydrate and protein, and high in dietary fiber.

Another intriguing difference between TRF and FMD is their difference in promoting the regeneration of intestinal cells. Only FMD but not TRF could increase the number of Lgr5^+^ cells and proliferating markers in the colon (Figure 6). While this observation is consistent with previous findings that intermittent caloric restriction with FMD can promote the regeneration and repair of the damaged intestinal epithelium in the IBD mouse model (18, 19), TRF appears to not be capable to promot this regeneration process. The difference between FMD and TRE in improving the regeneration of intestinal cells may explain why FMD has a moderately better effect than TRF in improving IBD in our model. Overall, the FMD group performed slightly better than the TRF group in improving DAI (Supplementary Figure 2), colon length (Figure 2A), number of colon crypt (Figure 2D), and spleen CD4^+^ T cells (Figure 3E). Collectively, it appears that TRF improves IBD mainly by reducing the inflammatory response of the intestine. Nevertheless, due to the potent anti-inflammatory activity of TRF and its metabolism-improving functions (17), it will be important in the future to further explore the intervention functions of TRF in many different types of immune-related disorders and other diseases.

## Data availability statement

The original contributions presented in this study are included in the article/Supplementary material, further inquiries can be directed to the corresponding author.

## Ethics statement

All animal experimental procedures were approved by the Institutional Animal Care and Use Committee (IACUC) guidelines of Shanghai Institute of Nutrition and Health, Chinese Academy of Sciences with an approval number SINH-2020-CY-1.

## Author contributions

YC and SS conceptualized and designed the study. SS performed the experiment. LC, MB, SW, XY, YL, XL, ZL, LZ, and XZ provided the technical assistance. ZW provided the editorial assistance. SS and YC wrote the manuscript and prepared the figures. All authors read and approved the manuscript.
