# Supplementary material for: Time-restricted feeding ameliorates dextran sulfate sodium-induced colitis via reducing intestinal inflammation
Source: Front Nutr. 2022 Dec 23;9:1043783. doi: 10.3389/fnut.2022.1043783 (PMC9822721; doi:10.3389/fnut.2022.1043783)
Supplement: Supplementary file 1 [file Data_Sheet_1.docx]

**Supplemental Figures**


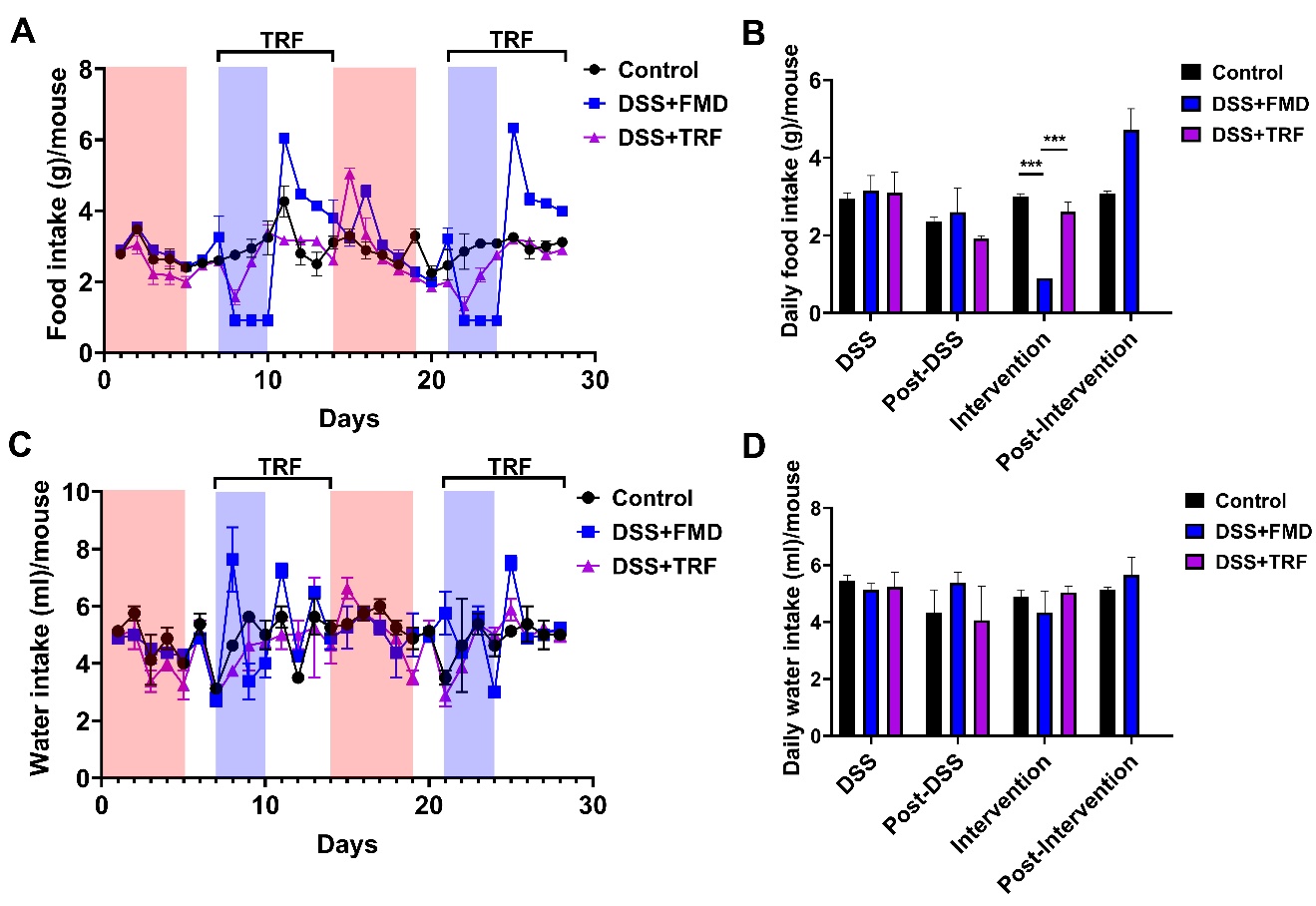
**Figure S1. Measurement of food and water consumption starting at the first day of cycle 2.**

1. Daily food intake of the mice.
2. Quantitation of daily food intake.
3. Daily water consumption of the mice.
4. Quantitation of daily water consumption.

Data are presented as mean ± SEM; *** P < 0.001.


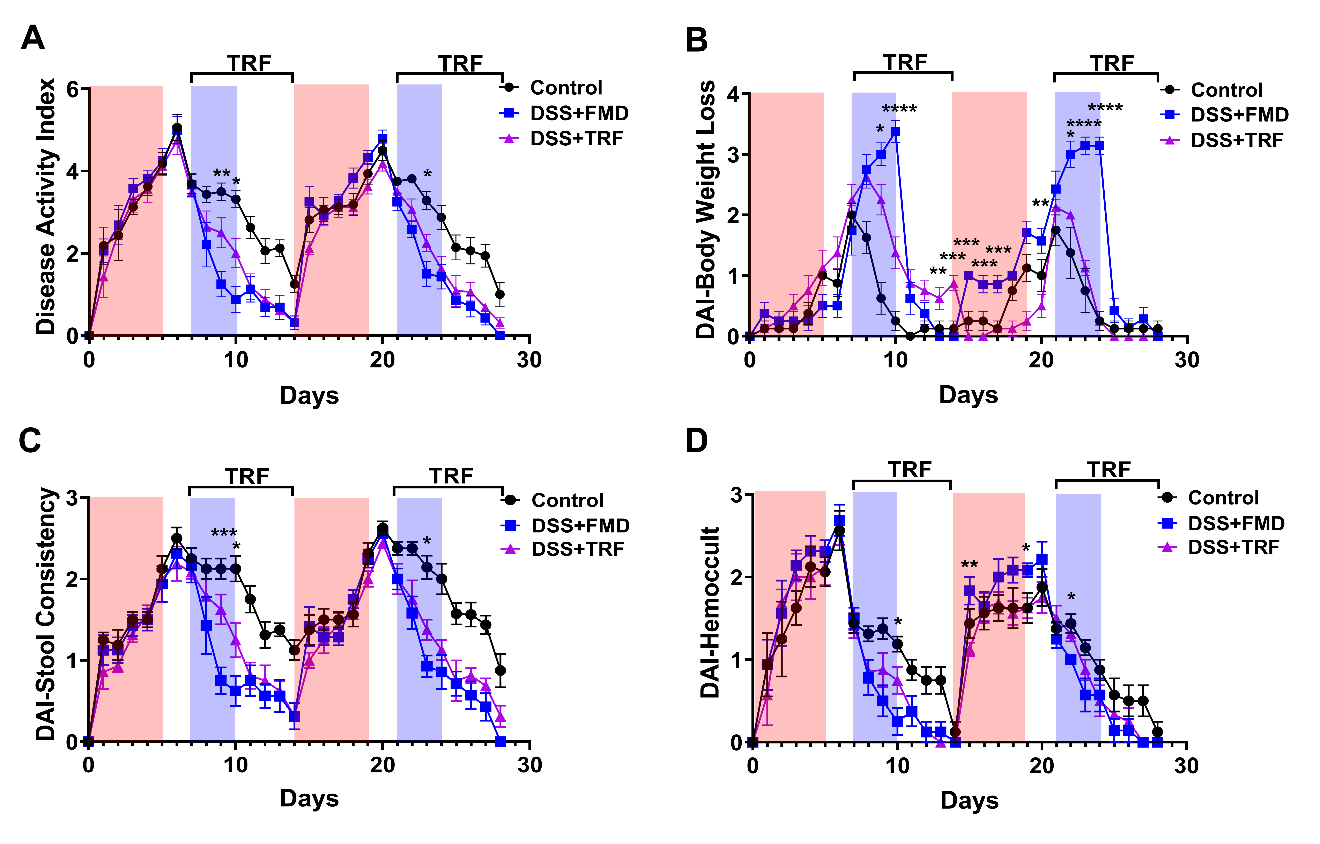


**Figure S2. FMD has a slightly better effect than TRF on decreasing DAI scores**

1. Cumulative DAI scores (excluding body weight loss score) that starting at the first day of cycle 2 (n = 8 for each group).
2. DAI scores of body weight loss.
3. DAI scores of stool consistency.
4. DAI scores of hemoccult test.

In A-D, the red shade denotes the duration of DSS treatment and the blue shade indicates the duration of FMD intervention, the duration of TRF is represented by the black bracket. Data are presented as mean ± SEM. The * sign represents the differences between the DSS+TRF group and the DSS+FMD group. *P<0.05, **P<0.01, ***P<0.001, ****P<0.0001.
